# Dihydropyridine Derivatives as Cell Growth Modulators In Vitro

**DOI:** 10.1155/2017/4069839

**Published:** 2017-04-03

**Authors:** Imanta Bruvere, Egils Bisenieks, Janis Poikans, Janis Uldrikis, Aiva Plotniece, Karlis Pajuste, Martins Rucins, Brigita Vigante, Zenta Kalme, Marina Gosteva, Ilona Domracheva, Astrida Velena, Tea Vukovic, Lidija Milkovic, Gunars Duburs, Neven Zarkovic

**Affiliations:** ^1^Latvian Institute of Organic Synthesis, 21 Aizkraukles Str., Riga LV-1006, Latvia; ^2^Laboratory for Oxidative Stress, Rudjer Boskovic Institute, Bijenicka 54, 10000 Zagreb, Croatia

## Abstract

The effects of eleven 1,4-dihydropyridine derivatives (DHPs) used alone or together with prooxidant anticancer drug doxorubicin were examined on two cancer (HOS, HeLa) and two nonmalignant cell lines (HMEC, L929). Their effects on the cell growth (^3^H-thymidine incorporation) were compared with their antiradical activities (DPPH assay), using well-known DHP antioxidant diludine as a reference. Thus, tested DHPs belong to three groups: (1) antioxidant diludine; (2) derivatives with pyridinium moieties at position 4 of the 1,4-DHP ring; (3) DHPs containing cationic methylene onium (pyridinium, trialkylammonium) moieties at positions 2 and 6 of the 1,4-DHP ring. Diludine and DHPs of group 3 exerted antiradical activities, unlike compounds of group 2. However, novel DHPs had cell type and concentration dependent effects on ^3^H-thymidine incorporation, while diludine did not. Hence, IB-32 (group 2) suppressed the growth of HOS and HeLa, enhancing growth of L929 cells, while K-2-11 (group 3) enhanced growth of every cell line tested, even in the presence of doxorubicin. Therefore, growth regulating and antiradical activity principles of novel DHPs should be further studied to find if DHPs of group 2 could selectively suppress cancer growth and if those of group 3 promote wound healing.

## 1. Introduction

Growth modulation, that is, proliferation induction or decline, is fundamental for cellular metabolic processes both in the health and in disease, as well in pharmaceutical interventions. Particularly regenerative medicine needs nontoxic proliferation inducers for cell, tissue, and organ regeneration. On the other side, proliferation inhibitors are necessary for the prevention and inhibition of uncontrolled growth of cancer cells. Recently [1] it was found that same 1-benzyl substituted 1,4-dihydropyridines (1,4-DHPs), activating SIRT1, are proliferation inhibitors in the cancer cells and on the contrary proliferation promoters in the wound healing. Direction of the search of the compounds acting in dual mode seems to be perspective.

Cellular redox signaling, including oxidative stress (OS) related events, is connected with genetic and epigenetic regulatory systems. Reactive oxygen species (ROS) and lipid peroxidation products are not only cytotoxic but may also perform and modulate signal transduction in cells. Accordingly, antioxidants (AOs) and radical scavengers may be considered as modifiers of cellular redox signaling, as well as genetic and epigenetic events, and thus 1,4-dihydropyridines being a group of synthetic antioxidants could be used for modulation of cellular redox signaling. Oxidative stress may have at least dual effects on cell proliferation and growth: anticancer-like effects as well as protumorigenic effects. The last ones are primarily related to induction of oxidative DNA lesions (8-OH-G) and consequential increase of DNA mutation frequency. These undesirable changes may, if not repaired, lead to genome instability and an increased rate of cellular proliferation [2]. Antineoplastic (anticarcinogenic, antitumorigenic) effects of OS have been closely linked to cellular processes of senescence and apoptosis, two major molecular mechanisms that counteract tumor development [3]. Which of these two actions will dominate depends on many factors including the metabolic status of the cell, as recently reviewed [4]. Accordingly, many AOs, for instance, curcumin [5], may be antineoplastic and cytotoxic by targeting mitochondria, affecting p53-related signaling and blocking NF-kappa B activation. A number of other curcumin targets include the aryl hydrocarbon receptor, cytochrome P450, glutathione S-transferase, serine/threonine kinases, transcription factors, cyclooxygenase, ornithine decarboxylase, nitric oxide synthase, matrix metalloproteinases, and tyrosine kinases. Some of these targets are characteristic also for DHPs antioxidant action [6].

Some of the amphiphilic compounds possessing self-assembling properties and forming nanoparticles in an aqueous medium could form stable liposomes [7–10] which are suitable as gene (pDNA) delivery agents in vitro, while the cytotoxicity and antiradical activity (ARA) of these amphiphilic 1,4-DHP derivatives were determined, too [10].

Biological activity of some of these compounds was previously studied (for antioxidant** diludine** ([11], see as cited in [6]), amphiphilic 1,4-DHP derivative, MDR modifier and suitable gene (plasmid DNA) delivery agent in vitro** K-2-11** [10], neuromodulator** AP-12** [12, 13], and also close compound** Z41-74** [14] (see also* Discussion* part)). However, physiological activity profile for most of mentioned compounds has not been still determined and published.

Presented work includes studies about a set of 11 original 1,4-dihydropyridine derivatives (comprising different substituents at positions 4, 2, and 6 or 3 and 5, containing neutral or cationic moieties, with diverse lipophilic or amphiphilic properties).

**Figure 1:**
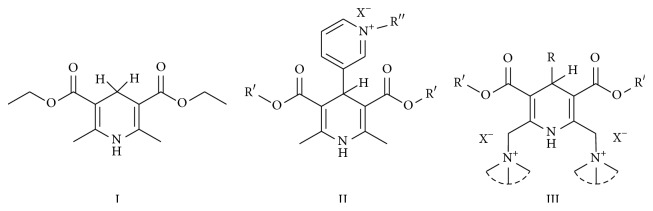
Core structures of studied 1,4-DHP derivatives (for details see Table 1).

The studied eleven DHP derivatives could be divided into 3 groups considering structure fragments (see Figure 1, Table 1):

1. 1.4-Unsubstituted 1,4-DHP (I, compound (1) in Table 1)
2. 1,4-DHPs comprising N-quaternized pyridine moiety at position 4 of the DHP ring (II, compounds (2)–(4) in Table 1)
3. 1,4-DHPs containing cationic onium methylene moieties at positions 2 and 6 of the DHP cycle (III, compounds (5)–(11) in Table 1) (in this set previously reported compound (12) (**Z41-74**) was included for more detailed analysis of relationships)

These DHPs were studied as potential cell proliferation modulators in two normal (human mammary epithelial cells HMEC and murine fibroblasts L929) and in two malignant cell lines (human osteosarcoma HOS and human cervical carcinoma HeLa). The effects of tested DHPs occurred if they were used alone or together with the well-known anticancer, prooxidant drug doxorubicin. Namely, doxorubicin causes long-lasting stimulation of ROS generation and OS in cancer cells and in cardiomyocytes [15]. Therefore, it is assumed that certain antioxidants (including DHPs) may influence the undesired side effects of doxorubicin, like cardiotoxicity. Some suggestions about DHP structure-activity relationships and selectivity on the above-mentioned cell lines are proposed.

## 2. Materials and Methods

All DHP derivatives (see further as listed in Table 1 and given in the* Results and Discussion* part) provided for the cell proliferation evaluation and used in this study have been synthesized in the Laboratory of Membrane active compounds of the Latvian Institute of Organic Synthesis (Latvian IOS).

Compound** AP-12** was obtained following an already reported method [16];** A2-15 ** was obtained according to procedure described by Makarova et al. [17]; compounds** K-2-11**,** IOS-10003**,** D-3-59-1,** and** K2-71** were obtained following an already reported method [10]; compound** IOS-10004** was obtained based on analogy by reported method [10]; compounds** V-1-32** and** V-1-41** were obtained according to procedure described by [18]; compound** IB-32** was obtained according to procedure described by [19].

### 2.1. Antiradical (Free Radical Scavenging) Activity (ARA)

ARA data were obtained spectrophotometrically using decoloration reaction ability with 1,1-diphenyl-2-picrylhydrazyl (DPPH) as a free radical scavenger [20], adapted for DHPs [10, 16]. An aliquot (0.5 mL) of the tested 1,4-DHP derivative solution in EtOH was added to 3 mL of freshly prepared DPPH solution in EtOH (0.1 mM). The final concentration of the tested compounds was 0.086 mM and the ratio of the tested compound and DPPH was equimolar. The solution was incubated for 30 min in the dark and changes in the optical density of solution were measured at 517 nm using a UV/Vis Camspec M501 spectrometer (UK). Each assay was performed in triplicate.

The scavenging activity was defined as the decrease in sample absorbance versus absorbance of DPPH standard solutions. Results were expressed as a percentage (%) of the DPPH free radical scavenging, which is defined by the following formula:

(1) $$\begin{array}{c} ARA\;\left(\;\%\;\right)=\frac{A_{control}-A_{sample}}{A_{control}}\times 100, \end{array}$$

where *A*_control_ is the absorbance of the standard solution of DPPH and *A*_sample_ is the absorbance value for the sample.

### 2.2. Basal Cytotoxicity Test

The Neutral Red Uptake (NRU) Assay was performed according to the standard protocol of [21] modified by NICEATM-ECVAM (Committee on the Validation of Alternative Methods (ICCVAM) of National Toxicology Program (NTP) Interagency Center for the Evaluation of Alternative Methods (NICEATM)) validation study [22]. The NRU cytotoxicity assay procedure is based on the ability of viable cells to incorporate and bind neutral red, a supravital dye. 3T3 (Mouse Swiss Albino embryo fibroblast) cells (purchased from ATCC®) (9000 cells/well) were placed into 96-well plates for 24 h in Dulbecco's modified Eagle's medium (DMEM) containing 5% fetal bovine serum and then exposed to the test compound over a range of eight concentrations (1000, 316, 100, 31, 10, 3, and 1 *μ*g/mL) for 24 h. Untreated cells were used as a control. After 24 h, the medium was removed from all plates. Then, 250 *μ*L of neutral red solution was added (0.05 mg/mL NR in DMEM, 24 h, preincubated at 37°C and then filtered before use through 0.22 *μ*m syringe filter). Plates were incubated for 3 h and then cells were washed three times with PBS. The dye within viable cells was released by extraction with a mixture of acetic acid, ethanol, and water (1 : 50 : 49). Absorbance of neutral red was measured using spectrophotometer multiplate reader (TECAN, Infinite M1000) at 540 nm. The optical density (OD) was calculated using the formula: OD (treated cells) *∗* 100/OD (control cells). The IC_50_ values were calculated using the program Graph Pad Prism® 3.0.

### 2.3. Estimation of LD_50_ from IC_50_ Values

Data from the in vitro tests were used for estimating the starting dose for acute oral systemic toxicity tests in rodents (mice, rat). The in vivo starting dose is an estimated LD_50_ value calculated by inserting the in vitro IC_50_ value into a regression formula: log LD_50_ (mM/kg) = 0.439log⁡IC_50_ (mM) + 0.621 [23]. The value is recalculated to mg/kg and compounds are evaluated in accordance with 4 toxicity categories [24]: category 1: LD_50_ ≤ 5 mg/kg (highly toxic); category 2: 5 < LD_50_ ≤ 50 mg/kg (moderately toxic); category 3: 50 < LD_50_ ≤ 300 mg/kg (slightly toxic); category 4: 300 < LD_50_ ≤ 2,000 mg/kg (practically nontoxic). Using an alternative in vitro method allows comparisons of possible toxicity of new compounds and selecting compounds for further study vastly reducing the number of animal experiments.

Radioactive thymidine assay was used for quantification of* cell proliferation modulation* properties of DHPs.

### 2.4. ^3^H-Thymidine Assay

To test the effects of DHPs on the growth of different types of cells in vitro we used the ^3^H-thymidine incorporation assay reflecting the DNA synthesis (i.e., the cell growth) of murine skin fibroblasts L929 cell line (NCTC clone 929 [L cell, L-929, derivative of Strain L] (ATCC CCL-1™)), human endothelial cells HMEC-1 (ATCC CRL-3243™), human cervical carcinoma HeLa (ATCC CCL-2™), and human osteosarcoma cell line HOS (ATCC CRL-1543™), which in vitro grows resembling osteoblast cells.

For the ^3^H-thymidine incorporation assay, the cells were seeded in microtiter plates (TPP, Swiss) at a density of 6 × 10^4^ cells/well and were treated with DHPs at three various stepwise concentrations: 1 *μ*g/mL, 10 *μ*g/mL, and 100 *μ*g/mL (approximately molar concentrations (~1 *μ*M, ~10 *μ*M, ~100 *μ*M) could be calculated from molecular mass data presented in the Table 1). Stock solutions of the compounds were obtained, diluting 5 mg of each compound in absolute ethanol to get concentration of 10 mg/mL. Some of the substances were not completely soluble; therefore in this case 5–10% DMSO were added.

After 1 h preincubation, ^3^H-thymidine (methyl-^3^H-thymidine, 25 Ci/mmol, Amersham) diluted with medium at a 1 : 25 ratio was added and the cells were cultured in humidified atmosphere containing 5% CO_2_ in DMEM culture medium supplemented with 5% fetal calf serum (Sigma, USA) for the following 24 h. After that, the cells were washed by cell harvester (Scatron, Norway) over a filter and the radioactivity of incorporated ^3^H-thymidine was detected in a beta-counter (Beckman LS 100C). Each group of cultures comprised four samples.

Control cells were equally cultured but without the presence of DHPs.

In the experiments with doxorubicin (Sigma) its stock solution concentration was 1 mg/mL (the 1 mg content diluted in 1 mL H_2_O). Three doxorubicin concentrations used were 0.1 *μ*g/mL, 0.5 *μ*g/mL, and 1 *μ*g/mL.

Dilution of stock solutions was made as follows.

Stock solutions in concentration 10 mg/mL were further diluted in DMEM to final concentrations 1, 10, and 100 *μ*g/mL. The rest of the solutions were stored at +4°C in plastic micro tubes with screw caps and used in next experiments.

The cells are plated in 96-well plates; cell cultures were incubated in DMEM media, containing 2.5% FCS, 2 × 10^6^ cells count in 6 mL DMEM (0.3 mL FCS) (each well of the plate was filled with 1–5 × 10^4^ cells (depending on the cell line) in 90 *μ*L cultured medium). After 4 h the cells were treated with the DHPs, while 1 h after that doxorubicin was added. The radioactive thymidine was added to cultures and left again for additional 24 h to incorporate ^3^H-thymidine, when the assay was performed on beta-counter (Beckman LS 100C).

Incubation was performed as follows.

Incubation and its duration (added volume)

1. cell culture → 4-5 h (100 *μ*L)
2. DHPs, DOXO → 24 h (100 *μ*L (50 + 50 *μ*L))
3. ^3^H TIM 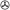 → 24 h (20 *μ*L)

Three identical repeated experiments were done for each treatment protocol of each cell line used.

## 3. Results

List of studied eleven 1,4-dihydropyridine derivatives, their structural formulas, molecular weight data, LD_50_ values in mg/kg (on NIH 3T3 cells), and antiradical activity data (DPPH assay, in %) (ARA) and data for previously reported compound** Z41-74** for more detailed analysis of relationships are given in Table 1.

The respective estimated LD_50_ values (see Table 1) for tested 1,4-DHP derivatives are different. Some of the listed compounds have the medium toxicity, some low according to 4 toxicity categories. Compounds** IB-32**,** AP-12**, and** K2-71** (in the lesser extent** K-2-11**,** D-3-59-1**) exerted toxicity, which could be classified as dangerous, while other compounds** diludine**,** A2-15**, and** V-1-41** could be classified as nontoxic DHPs.

### 3.1. Antiradical Activity (ARA) of Various DHPs

Relative antiradical activity (ARA, expressed in %) was determined using DPPH method [10, 20], for DHPs chosen upon the results of cytotoxicity assays to be tested further in the cell growth experiments.

Thus, a wide range of ARA, from relative high and significant ARA for compounds** IOS-10003**,** IOS-10004**,** V-1-41**, to the absence of ARA, as for compounds** AP-12**,** A2-15**, and** IB-32**, were revealed (see Table 1).

Medium ARA values were obtained for compounds** K-2-11** and** diludine**, 40.5 and 39.5%, respectively.

One can notice that all studied compounds possessing cationic (onium: trimethylammonium, methylcycloalkylammonium, or pyridinium) substituents at 2 and 6 methylene of the DHP cycle have more or less significant ARA. In case of 2,6-methylmorpholiniummethylene substituents (compounds** IOS-10003** and** V-1-32**) the highest ARA are detected (95% for each other).

Electron-donating substituents' containing pyridiniomethylene moieties at positions 2 and 6 of the 1,4-DHP diminished the ARA (compound** D-3-59-1**), while DHPs have shown ARA also in case of absence of electron-withdrawing cationic groups at positions 2 and 6 of the 1,4-DHP cycle (**diludine**). It should be mentioned here that corresponding 4-phenyl analogue was practically inactive (see [6]).

As showed in the previous studies, compound** Z41-74** stimulated HOS cell growth [14]. This compound has trimethylammoniomethylene substituents at positions 2 and 6 of the DHP cycle and is relative structural analogue of compounds** V-1-32**,** IOS-10003** and** IOS-10004** with saturated cationic heterocyclic moieties at positions 2 and 6 of the DHP cycle. However compound** Z41-74** possesses lower antiradical activity (16%), perhaps due to aliphatic (uncyclic) trimethylammonio part in molecule.

There are 3 compounds with cationic N-quaternized pyridinium moiety at position 4 of the 1,4-DHP cycle,** A2-15** and** AP-12**, that have shown perspective as bifunctional growth modulating agent compound** IB-32**, lacking ARA. These compounds lack cationic moieties at position 2 and 6 capable of forming labile hydrogen atom.

A group comprising compounds** V-1-32** and** V-1-41** (see Table 1) having both 2,6-pyridiniomethylene substituents, but with different variations at position 4 of the 1,4-DHP cycle, gave relatively high values of ARA (68.8% and 70.7% correspondingly). Therefore substituent in position 4 seems to have less decisive effect than do variations at positions 2 and 6 of the 1,4-DHP molecule. Their influence on normal and on tumor cells was less manifested; there was no difference in case of normal cell growth and in case of** V-1-32**, either on HOS or on HeLa cells growth (except 100 *μ*g/mL concentration which slightly retarded cell proliferation). Nevertheless, compound** V-1-41** increased growth of HeLa cells and HOS cells indicating it could be used to study further altered mechanisms of the malignant cell growth.

These compounds have shown low toxicity (1706 mg/kg and >2000 mg/kg correspondingly) and did not suppress growth of any cell type even at the concentration 100 *μ*g/mL.

These compounds have structure similarities with compounds** IOS-10003** (versus** V-1-32**) and** K-2-11** (versus** V-1-41**) that also share similar biological activities, although one can notice some minor differences.

### 3.2. Dependence of the Cell Growth Regulating Activities of the DHP Derivatives on Their Structure and Concentrations Used

**Figure 2:**
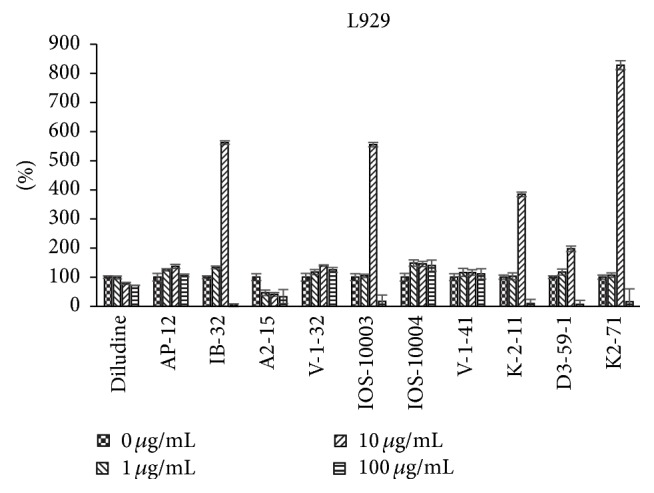
Dependence of proliferation modulation activity (expressed as ^3^H-thymidine incorporation, in percentage (*y*-axis) of the respective, untreated control) on chemical structure and concentration of eleven DHP compounds on L929 cells.

**Figure 3:**
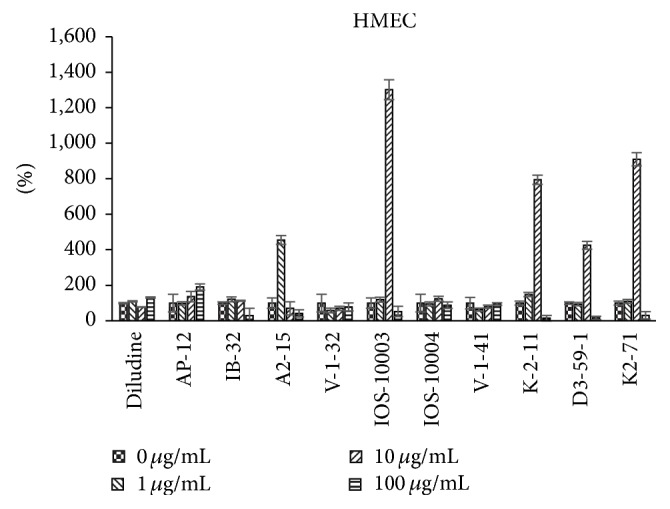
Dependence of proliferation modulation activity (expressed as ^3^H-thymidine incorporation, in % of control, *y*-axis) on chemical structure and concentration of eleven DHP compounds on HMEC cells.

**Figure 4:**
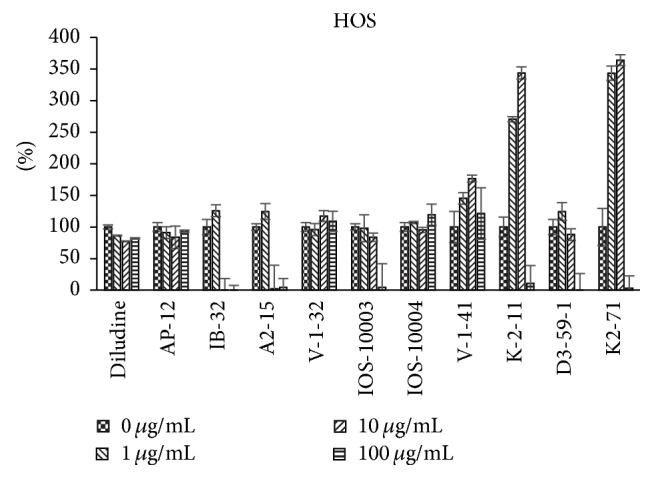
Dependence of proliferation modulation activity (expressed as ^3^H-thymidine incorporation, in % of control, *y*-axis) on chemical structure and concentration of eleven DHP compounds on HOS cells.

**Figure 5:**
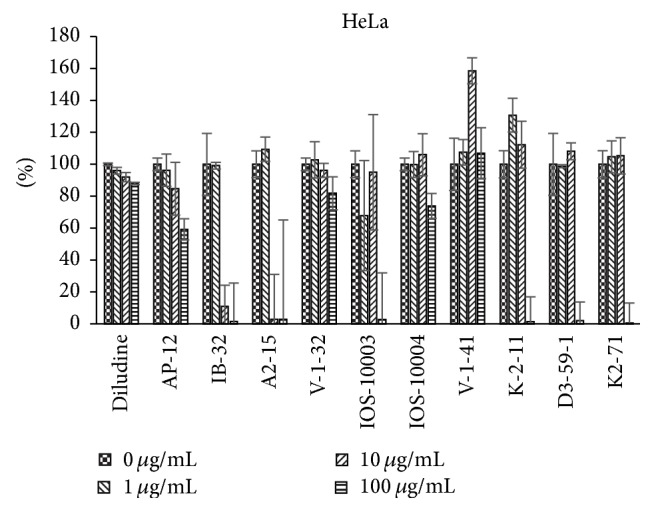
Dependence of proliferation modulation activity (expressed as ^3^H-thymidine incorporation, in % of control, *y*-axis) on chemical structure and concentration of eleven DHP compounds on HeLa cells.

The data regarding 1,4-DHPs growth modulating activities were obtained using the ^3^H-thymidine incorporation assay reflecting the cellular DNA synthesis (i.e., the cell growth). Different types of cells in vitro were used: murine skin fibroblasts L929 cell line (Figure 2), human endothelial cells HMEC (Figure 3), human osteosarcoma cell line HOS, which in vitro grows resembling osteoblast cells (Figure 4), and human cervical carcinoma HeLa (Figure 5). Concentration/activity and structure/activity relationship of tested 11 DHP derivatives are presented in Figures 2–5. In Figures 2–5 on ordinate axis the value of proliferation activity as percentage of the untreated controls is given. Three stepwise concentrations were used: 1 *μ*g/mL, 10 *μ*g/mL, and 100 *μ*g/mL. Since the different experiments had different control levels of incorporation to compile the data of 3 experiments it was calculated. Data are presented as mean ± SD.

**Figure 6:**
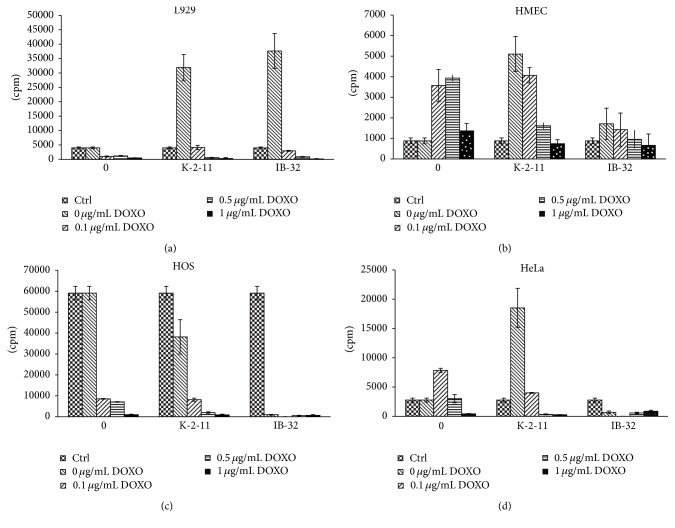
(a) Proliferation modulation activity of DOXO (columns 0) and of DHP derivatives** K-2-11** and** IB-32** (10 *μ*g/mL) alone and in the presence of DOXO on L929 cells (expressed as ^3^H-thymidine incorporation, in CPM, *y*-axis). Control cells (Ctrl) without DOXO (0 *μ*g/mL) are presented in the 1st bar of each column. First column (0): control cells treated with DOXO, without DHPs. Added DOXO concentrations were expressed as *μ*g/mL: 0.1 *μ*g/mL, 0.5 *μ*g/mL, and 1 *μ*g/mL. Results were expressed as mean values of counts per minute (CPM) ± SD. (b) Proliferation modulation activity of DOXO (columns 0) and of DHP derivatives** K-2-11** and** IB-32** (10 *μ*g/mL) alone and in the presence of DOXO on HMEC cells (expressed as ^3^H-thymidine incorporation, in CPM, *y*-axis). (c) Proliferation modulation activity of DOXO (columns 0) and of DHP derivatives** K-2-11** and** IB-32** (10 *μ*g/mL) alone and in the presence of DOXO on HOS cells (expressed as ^3^H-thymidine incorporation, in CPM, *y*-axis). (d) Proliferation modulation activity of DOXO (columns 0) and of DHP derivatives** K-2-11** and** IB-32** (10 *μ*g/mL) alone and in the presence of DOXO on HeLa cells (expressed as ^3^H-thymidine incorporation, in CPM, *y*-axis).

Further proliferation modulation activity of some DHPs is compared with DOXO activity (see Figure 6).

All tested DHPs exerted cell type-dependent and concentration dependent effects, as quantified by the ^3^H-thymidine incorporation (DNA synthesis) of the cultured cells (see Figures 2–5). Nonlinear dependence of effect on the concentrations of compounds used was observed. Medium concentrations of DHP derivatives showed usually the most pronounced effects, except usually cytotoxic effects of the highest concentrations (see Figures 2–5). The more effective one was concentration 10 *μ*g/mL (for compound** K-2-11**, on all four cell lines, for** IOS-10003,** on L929 and HeLa cells). The highest concentration 100 *μ*g/mL was however manifold less effective than 10 *μ*g/mL or even cytotoxic (in the case of** K-2-11**, on HeLa cells).

While all 4 substituted 1,4-DHPs, except** diludine**, were completely abolishing the ^3^H-thymidine incorporation by L929 cells if used at 100 *μ*g/mL concentration indicating even cytotoxic activities of DHPs, the** K-2-11**,** D3-59-1,** and in particular** IB-32** and** IOS-10003** on the other hand strongly enhanced the growth of these fibroblasts, but only if used at 10 *μ*g/mL concentration. However, compound** IB-32** did not show such growth enhancing effect for any other cell line used. Actually compound** IB-32** was toxic at 10 *μ*g/mL concentration for HOS and for HeLa cells. Opposite to that, compound** K-2-11** enhanced the growth of every cell line tested, if used at 10 *μ*g/mL concentration. The most pronounced stimulating effect of compound** K-2-11** was observed for HMEC endothelial cells (Figure 3), while for HeLa cells it was more or less enhancing (Figure 5). It should also be mentioned that growth stimulating effects of compound** K-2-11** obtained at 10 *μ*g/mL concentration were changed into strong suppression, that is, cytotoxicity in case of 100 *μ*g/mL concentration of** K-2-11**.

The most similar effects to those of derivative** K-2-11** were effects of compound** K2-71**, with only exception of the even higher efficiency of compound** K2-71** in case of L929 cells stimulation (Figure 2). Compound** K2-71** was efficient DHP stimulating L929, also HOS and HMEC cells (Figures 3 and 4) even more than compound** K-2-11**, while for all cell lines tested it was toxic if used at 100 *μ*g/mL concentration.

Interestingly, the well-known DHP antioxidant** diludine** did not exert as strong effects as did the other DHPs; even in case of 100 *μ*g/mL concentration** diludine** did not show strong toxicity as did the other DHPs tested.

As in case of other physiological activities (antihypertensive, anticancer, etc.), substituents at positions 4, 2 and 6, and 3 and 5 of the 1,4-DHP cycle and also the lipophilicity of the molecule have important effects on the antiproliferative activity of 1,4-dihydropyridine derivatives. Regarding structure-activity relationships, present work claims the importance of hetaryl substituents at position 4 of the DHPs (compound** IB-32**, see Table 1) (analogous as given in [25]). Active are aryl (phenyl-) substituents too (see Table 1).

The length of alkyl chains of substituents at positions 3 and 5 may be shorter (methyl or ethyl esters), medium (propoxyethoxy ester), or prolonged (till C-12: dodecyl ester), substituents at positions 2 and 6 may be charged bilaterally. The charge in position four seems to be important, while activity is present both for lipophilic compounds (low activity for** diludine**, see Table 1), and, especially, for amphiphilic compounds too, analogues of** K-2-11**.

Compound** V-1-32** containing N-methylmorpholiniomethylene substituent at positions 2 and 6 analogue comprising 4-[2′-difluoromethoxyphenyl] moiety and shorter (methyl) alkyl chains in ester groups at substituents 3 and 5, as well as** V-1-41** (which is structural analogue of known compound** Z41-74** comprising methoxycarbonyl group (**V-1-41**) at position 4 of the 1,4-DHP cycle instead of phenyl group (**Z41-74**)) with pyridiniomethylene substituents at positions 2 and 6, only insignificantly influences normal and tumor cell proliferation.

On the contrary, DHP derivative** K-2-11** possessing ARA properties [10] significantly increased proliferation of HOS cells at 10 *μ*g/mL concentration. The data obtained for HeLa cells are contradictory: 10 *μ*g/mL concentrations increased HeLa cells proliferation almost 10 times, but some experiments revealed absence of statistically significant increase of HeLa cells proliferation. Compound** K-2-11** increased also proliferation of normal cells (cell lines HMEC and L929). At the concentration 100 *μ*g/mL the growth of cells was suppressed, including also tumor cell lines HeLa and HOS. Cytotoxicity on normal cells is low: NIH 3T3 is 1482 mg/kg (see Table 1).

Compound** K2-71** containing 3-methylpyridiniomethylene substituents at positions 2 and 6 is a close relative (homologue) of compound** K-2-11**. It has medium ARA (~39%), low cytotoxicity, and enhanced growth of normal cells at concentration of 10 *μ*g/mL, stimulating also HOS cells (at 1 and 10 *μ*g/mL), but not HeLa cells, while at 100 *μ*g/mL growth of all cell types was suppressed.

Compound** IOS-10003** is intriguing compound: it has high ARA (~95%) [10]. It is effective proliferation promoter at 10 *μ*g/mL for the L929 cells (Figure 2) and in HMEC cells (Figure 3). At 100 *μ*g/mL concentration proliferation of L929 and HMEC was suppressed significantly similar to malignant HOS and HeLa cells.

Compounds** V-1-32** and** V-1-41** have similar and also different structural parameters comparing to** IOS**-**10003** (versus** V-1-32**) and** K-2-11** (versus** V-1-41**), so also properties are at the same time similar in some sense, but one can also notice difference. Compounds** V-1-32** and** V-1-41** possess high ARA (95.1 and 70.7% correspondingly); nevertheless their influence on normal and tumor cells growth was less manifested. There was no effect on normal cells growth; and in case of compound** V-1-32** there was also no influence on HOS and HeLa cell growth. However, compound** V-1-41** increased the growth of HeLa and HOS cells. Both compounds have low cytotoxicity (1706 mg/kg and >2000 mg/kg correspondingly). They did not suppress growth of any cell line even at concentration 100 *μ*g/mL.

Similarly, compound** A2-15** (one representative from compounds group II (**A2-15**,** AP-12**, and** IB-32**, but without ARA)) was mainly inert regarding studied cell lines. Concerning compound** AP-12** one can mention its enhancing effect on HMEC cells growth and diminishing effect for the growth of HeLa cells at 10 *μ*g/mL and 100 *μ*g/mL concentrations.

On the contrary,** IB-32** revealed quite unique properties, enhancing growth of normal cells (especially L929) and sharp diminishing or completely stopping the growth of HOS and HeLa cells (at concentrations 10 *μ*g/mL and 100 *μ*g/mL). For L929 cells activity of compound** IB-32** was remarkable in the same activity range as for compound** K-2-11** (Figures 2 and 6(a)), being more efficient with DOXO concentration 0.1 *μ*g/mL.

The activities of remaining 5 compounds (**V-1-32**;** V-1-41**;** IOS-10004**;** A2-15**;** AP-12**) only insignificantly prevailed over control levels (without DHPs addition). Accordingly, we can define following groups of compounds concerning their influence on tested cell cultures:

1. Compounds significantly enhancing growth of cultured cells in case of
  (1a) Both normal and tumor cells:** K-2-11** (ARA 39.5%)
  (1b) Only normal cells: growth of tumor cells may be suppressed:** AP-12** (lacked ARA),** IB-32** (also lacked ARA), and** IOS-10003** (ARA 95.1%)
  (1c) Only tumor cells:** V-1-41** (ARA 70.7%)
2. Compounds revealing minor deviations on normal and tumor cells growth:** V-1-32** (ARA 95.1%),** IOS-10004** (ARA 54.1%),** A2-15** (ARA close to 0%), and** AP-12** (ARA close to 0%)

### 3.3. Proliferation Modulation Activity of DHPs in Combination with Doxorubicin

The anticancer drug doxorubicin (DOXO) was used at ranging concentrations: 0.1 *μ*g/mL, 0.5 *μ*g/mL, and 1 *μ*g/mL to test additional effects of compounds** K-2-11** and** IB-32** (see Figures 6(a)–6(d)). In the experiments with DOXO (see Figures 6(a)–6(d)) final concentration of both 1,4-DHP derivatives (**K-2-11** and** IB-32**) was 10 *μ*g/mL. Both compounds are representatives of 2 different groups of DHPs studied (**IB-32**: II group,** K-2-11**: III group).

Addition of DOXO itself reduced the growth of most cells (not significantly in case of HMEC cells), if used at high concentrations, while it surprisingly enhanced both normal cell line HMEC and cancerous cell line HeLa if used at lower concentrations.

On the contrary, the growth enhancing effects of compound** IB-32** were more selective; that is, they were observed only for normal cell lines L929 and HMEC, while** IB-32** entirely retarded growth of tumor cells HOS and HeLa. Compound** K-2-11** alone stimulated growth of normal (L929 and HMEC) cell lines but also of tumor (HeLa) cells; in case of HOS cells reduction of the cell growth was observed.

## 4. Discussion

### 4.1. Is Growth Regulating/Antiproliferative Activity Related to Antioxidant and/or Antiradical Properties of DHP Compounds?

Previously ARA was found only for a part of the 11 compounds set: for** diludine** (see [6]) and** K-2-11**,** Z41-74** [10, 14].

Comparison of proliferation modulation direction and strength of cationic moieties containing compounds** Z41-74** (see Table 1), compound, relative to** K-2-11**,** IOS-10003** [10], and** V-1-32** and** V-1-41** (see Table 1), and their dependence on ARA or AOA of these compounds was performed.

Compound** Z41-74** increased HOS cells growth attenuating lipid peroxidation effects [14]; it has low antiradical activity (16.1%).

Compounds having almost identical ARA had however different effects on cell proliferation. Archetype of DHP compounds, Hantzsch ester** diludine**, which is claimed as possessing remarkable AOA and ARA (ARA ≈ 41% in this study, see also appropriate citations in [6]) revealed almost inert attitude to normal and tumor cell proliferation. Compound** K-2-11** has almost identical ARA (ARA ≈ 40%); nevertheless it had significant influence on cell proliferation (HMEC, L929, HOS, and also HeLa). This may indicate that antioxidant properties of mentioned compounds (and probably other DHPs) are not the most relevant ones for their growth regulating effects.

Compound** IOS-10003** has high ARA (93.9%, see Table 1 and text of Section 3.2.); nevertheless it in general had very poor influence on normal and on tumor cells proliferation. Its effects were concentration dependent. So, it increased HMEC cell proliferation only at concentration 10 *μ*g/mL and decreased L929 cells proliferation at 10 *μ*g/mL and 100 *μ*g/mL concentration. It also slightly reduced HOS cells proliferation at all studied concentrations. Nevertheless influence on HeLa cells was more remarkable; at 10 *μ*g/mL concentration, proliferation rate was increased more than twice, while at 100 *μ*g/mL HeLa proliferation was stopped. At 100 *μ*g/mL for all cell types a decrease of proliferation was noticed.

We assume that hormetic redox signaling that is influenced by cellular stress could be important for the biological effects of DHPs, causing their biphasic dose-response relationship, manifested by low-dose stimulation and a high-dose inhibition of the cell growth, which seems to be true for most antioxidants [26, 27].

However, it seems there is no obvious relationship of ARA and influence of DHPs on cell proliferation, and the probability of the prediction of proliferation/regeneration inhibition or stimulation by DHPs using AOA or ARA activity as a criterion is the open question.

According to the ^3^H-thymidine incorporation data obtained using 11 DHPs to treat 4 cell lines we can conclude that the most effective growth enhancing DHP was** K-2-11**, which enhanced the growth of all cell lines, usually for 3-fold, being the most effective in case of HMEC cells (8-fold increase) and the least effective in case of HeLa cells (only about 20% increase). The highest growth enhancing capacity of** K-2-11** was also supported by its efficiency even at 1 *μ*g/mL concentration. Interestingly, similar to** K-2-11**,** K2-71**,** D3-59-1**, and** IB-32** had strongest growth enhancing effects exerted at 10 *μ*g/mL concentration, which was even stronger than in case of** K-2-11**, but just of one cell line for each of these DHPs. Hence,** K2-71** and** D3-59-1** were the most potent growth stimulators for the HMEC cell line, while** IB-32** enhanced only L292 murine fibroblasts. While it is not likely that such a specificity of IB-32 for L929 cells was due to the murine origin of these cells, we can assume that it rather reflects certain fibroblast cell growth enhancing capacity of** IB-32**. To verify this assumption additional fibroblast cell lines should be tested, since such an effect might be relevant for the possible use of** IB-32** to promote the wound healing, yet bearing a risk of the scar formation. It is certainly interesting that** K2-71** did not stimulate only the growth of L929 fibroblasts, so further comparisons between these two DHPs might help elucidating their activity principles.

The fact that most of the growth enhancing DHPs had strongest effects for human endothelial cells (HMEC) might indicate their possibly beneficial effects to support recovery of the damaged blood vessels or the neovascularization. That could be especially valuable for the promotion of the wound healing but could perhaps represent certain risk for the enhancement of the tumor neovascularization; therefore such effects should be further studied, too.

However, it is certain that none of these DHPs bears significant risk for enhancement of the cancer cell growth because none of them supported HeLa cell growth, while they all entirely abolished the growth of all cell lines tested if used at 100 *μ*M concentration, except** diludine**. The growth enhancement of human osteosarcoma cells (HOS) observed upon** K-2-11** and** K2-71** does not necessarily have to be negative, because HOS cells are considered to be the osteoblast-like cells and are therefore used often to evaluate the growth of bone cells.

Since most of the DHPs used might be considered also as potential antioxidants, while their effect of the cell growth is cell type and concentration dependent, it is possible to assume that they could affect the cellular redox signaling, either directly or indirectly affecting the redox signaling pathways. Of particular interest for further studies would be analysis of potential interference of DHPs with lipid peroxidation, notably with 4-hydroxynonenal (HNE), which is considered to act as second messenger of free radicals and as growth regulating factor showing effects similar to the effects of the DHPs tested [4, 28–30].

It could be proposed that there can be also different additional factors (binding with some nuclear factors and/or receptors, modulation of gene and protein expression) relevant for the results obtained. Thus, some water-soluble 1,4-dihydropyridine derivatives without Ca^2+^-antagonist activity, having proteolysis promoting activities, upregulate Psma6 mRNA expression in kidneys of intact and diabetic rats [31].

It should be mentioned also that in [32] instead of prooxidant toxicity of DOXO new alternative mechanism of doxorubicin antitumor effect is proposed, notably enhancement of de novo synthesis of ceramide, which in turn activates transcription factor CREB3L1. DOXO stimulates proteolytic cleavage of membrane-bound precursor of CREB3L1 by Site 1 Protease and Site 2 Protease, allowing the NH(2)-terminal domain of CREB3L1 to enter the nucleus and activate transcription of genes which encode inhibitors of the cell cycle, including p21. As mentioned above [31], water-soluble DHPs have proteolysis promoting activity. This property as one of the possible parts in cell growth regulating mechanism further should be examined and verified (maybe it is not only the case for water-soluble DHPs) for proliferation modulating DHPs tested in present study.

### 4.2. The Sensitivity (or Selectivity) of the Used Cell Lines toward Various DHP

The sensitivity (or selectivity) of the two normal cell lines (human endothelial cells HMEC and murine fibroblasts L929) and the two cancerous cell lines (human osteosarcoma HOS and human cervical carcinoma HeLa) toward proliferative/antiproliferative activity of DHPs both when used alone or in combination with DOXO appeared to be cell type different.

Namely, if the results obtained are analyzed according to the cell type used, it could observed that the most sensitive ones for the growth enhancing effects of the DHPs were human endothelial cells HMEC, while DHPs had hardly any stimulating effects for human cervical carcinoma cells HeLa.

Using L929 cell culture (Figure 2), it was found that compound** K-2-11** stimulates proliferation rate about 8 times. In case of** IB-32** (10 *μ*g/mL) the stimulation is about 9.5 times. In presence of 0.1 *μ*g/mL DOXO stimulation of cell proliferation was inhibited. It is also worth mentioning that** IB-32** has shown growth stimulating effects only for L929 murine fibroblasts, while it was the most effective DHP suppressing the growth of HOS and HeLa cells even if used at 10 *μ*g/mL concentration, unlike the other tested 1,4-DHP derivatives which were effective only at 100 *μ*g/mL concentration.

In case of HMEC cell line (see Figure 6(b)) stimulation of proliferation was observed mainly for compound** K-2-11**, as well as in presence of DOXO at 0.1 *μ*g/mL.

As shown by Figure 6(c), compound** IB-32** inhibited HOS cell proliferation better than DOXO at 0.1 *μ*g/mL and even 0.5 *μ*g/mL. Therefore, this compound seems to be very potent proliferation inhibitor and perspective anticancer drug. Compound** K-2-11** per se partially inhibits HOS cells proliferation and enhances inhibition of HOS cells proliferation in combination with 0.5 *μ*g/mL DOXO.

Compound** K-2-11** stimulated proliferation (Figure 6(d)) of HeLa cells (both if used alone, without DOXO, and if used in the presence of 0.1 *μ*g/mL DOXO). That makes it less likely to be proposed as anticancer proliferation modifier. Surprisingly, DOXO too at 0.1 *μ*g/mL stimulated proliferation rate.

On the contrary compound** IB-32** inhibited proliferation of HeLa cells both by itself and in the presence of DOXO.

### 4.3. Revealing Physiological Effects of Tested DHPs in relation to Their Growth Regulating Effects

Our present data are complementary to those previously obtained and to the results of other researchers concerning antioxidant, antiradical properties and growth regulating (proliferation modifying, both of normal and of cancer cell lines), as well as anticancer, MDR reversing, and other activities of studied DHP derivatives and their close analogues.

Growth regulation through redox signaling seems however not the only possible activity principles of the DHPs, although some other growth regulating mechanisms (apoptotic and antiapoptotic) could be also influenced by DHPs active as proliferation modulating agents. Namely, it was shown that the type of substituents at positions 1 and 4, 2 and 6, or 3 and 5 and also the lipophilicity of the molecule have substantial effects on the proliferation modulation and anticancer activity of 1,4-dihydropyridine derivatives [1].

Antioxidant** diludine** enhances growth performance of domestic animals, poultry, and fish; therefore it is claimed as functional growth enhancer in vivo (see [6]). However,** diludine** was until now never examined in cell proliferation experiments (on these four cell lines), so these data indicated it is probably metabolized/activated to become a growth promotor in vivo.

**Diludine** and water-soluble dihydroisonicotinic acid derivatives reveal antimutagenic and anticlastogenic properties and accelerate repair of oxidant and ionising radiation generated DNA damage (see Duburs et al.'s [33]).

Three water-soluble 1,4-dihydroisonicotinic acid derivatives were tested using ^3^H-thymidine incorporation and trypan blue assay for liver cells growth and viability [34]. All three substances caused a dose-dependent decrease in ^3^H-thymidine incorporation, but in different concentration ranges. Classical antioxidant Trolox caused very rapid decline in ^3^H-thymidine incorporation. Thus, depending on the structure and concentration, the cited 1,4-DHP derivatives variously affected thymidine incorporation, cell proliferation, and growth, connected with OS and other metabolic influences. Maybe further derivatization of the tested DHPs (**AP-12** and** IB-32**) with water-soluble substituents could be worthwhile. Compounds** K-2-11** and** K2-71**, as well as** IOS-10003**,** IOS-10004**, and** A2-15** have water-soluble properties.

Dual effects of a water-soluble 1,4-DHP compound (sodium 3,5-bis-ethoxycarbonyl-2,6-dimethyl-1,4-dihydropyridine-4-carboxylate) in X-irradiated* L5178Y cells* (*murine lymphoma sublines*, double strand break (DSB) repair competent* LY-R* and radiosensitive* LY-S* cells) are reported by Dalivelya et al. [35]. Decreased fixation of radiation inflicted DNA damage by increasing the rate of DNA repair and enhancing the efficiency of checkpoint control were postulated as activity principles of this DHP. However direct confirmation of this assumption is necessary. Ryabokon et al. [36] found that 1,4-DHP derivative reduces DNA damage and stimulates DNA repair in human cells in vitro.

*Amphiphilic* DHP compound** K-2-11** is claimed to be a multidrug resistance (MDR) reverser [37]. MDR often develops in cancer cells to different chemotherapeutic drugs and is essential factor in the failure of various chemotherapies [25], including those based on DOXO. In the last two decades some 1,4-DHPs and structurally related compounds were discovered and approved as effective reversers of resistance to doxorubicin, daunomycin, vinblastine, and vincristine, as other anticancer drugs [25, 38, 39]. The presence of a hetaryl group at position 4of DHP is claimed as effectively increasing MDR-inhibiting activity [25]. MDR modulating activity was found for near DHP analogues, thieno[2,3-b]pyridines too [40].

Compound** K-2-11** was tested [37] on MDR1-expressing mouse lymphoma cells and their parental control.** K-2-11** enhanced the cytotoxic effects of doxorubicin, both in the MDR and in parental cell line, while** K-2-11** alone did not affect cell viability. Our data however suggest that compound** K-2-11** could per se modulate proliferation activity, as well in the presence of DOXO (in HMEC and HeLa cells, see Figures 6(b) and 6(d)). Compound** K-2-11** also acted as an antioxidant, reducing the cellular generation of reactive oxygen species (ROS). It is assumed that 1,4-DHP derivative** K-2-11** blocks P-gp activity; thus cancer cells stay more chemosensitive due to DOXO retention in the cells. Compound** K-2-11** could suppress also increase of ROS (caused by DOXO and further doxorubicin semiquinone radical initiated reduction of molecular oxygen and generation of superoxide radical, which initiates ROS production chain reaction), preventing in this way NF-*κ*B activation that could consequently lead to a normal expression of MDR1 and antiapoptosis genes, restoring chemosensitivity of cancer cells.

The ability of compound** K-2-11** to modulate the growth the of cells found on MDR1-expressing mouse lymphoma cells and their parental control [37] was also confirmed in current study (see Table 1, Figures 6(a)–6(d)). Unfortunately** K-2-11** several times enhanced the HeLa cell growth, thus reducing its attractiveness to some extent (Figure 6(d)).

A novel* amphiphilic (lipophilic) *1,4-dihydropyridine derivative** AP-12** (see Table 1) was studied in the present article as proliferation modifier in the above-mentioned cell systems. Recently, Jansone et al., 2016 [13], have described memory-improving, anxiolytic effects of this compound in transgenic AD model (TgAPPSweDI) male mice. It was also shown [12] that this compound crosses the blood-brain barrier and blocks neuronal (neuroblastoma) and vascular (vascular smooth muscle cell line) calcium channels, exerting Ca^2+^ antagonistic properties, and changed brain protein expression (as postsynaptic membrane protein Homer-1), with a particular focus on those of the GABAergic system, and improved behavior. Previously direct correlation between the length of the alkyl chain substituent at structurally related N-quaternized 4-*β*-pyridyl-l,4-dihydropyridines and their improved “membranotropic” effects was noticed, such as incorporation in the liposomal membranes and bilayer fluidity could be one of essential activity principles of this DHP [41].

Compound** Z41-74** (as well as another* lipophilic* DHP compound neuroprotectant** cerebrocrast**) was previously tested for the influence on HOS (human osteosarcoma) cell line [14]. Both compounds** Z41-74** and** cerebrocrast** caused increased metabolic rate and growth of these cells, even attenuating suppressive effects of lipid peroxidation. Herewith we must say that HOS cells are known not only as sarcoma, but also as a model osteoblast cells, due to their growth features in vitro.

It must be also mentioned that recently SIRT1 activation and promotion of wound healing (proliferation) of normal cells and contrary suppression of tumor cell proliferation were found for some lipophilic 1-benzyl substituted 1,4-DHPs [1], similar to our data obtained concerning N-unsubstituted dually acting DHP compound** IB-32**.

Antihypertensive DHPs are structural analogues of compounds surveyed in the present study as proliferation modifiers. Antiproliferative effect for these compounds was reported as structure (substituent type and length, stereochemistry of substances) and concentration dependent, as well as being connected with lipophilicity parameters. Thus,* lipophilic* DHP calcium channel blockers** lacidipine** and** amlodipine** reduced carotid intima-media thickness by decreasing proliferative effect of oxidized low-density lipoprotein (ox-LDL) (antiproliferative effect against proproliferative effect of ox-LDL), whereas** (*S*-)-amlodipine** had no antiproliferative effect. ROS-MAPKs (Mitogen Activated Protein Kinases) pathway might be involved in the mechanism [42, 43]. Antihypertensive DHP drug** azelnidipine** (AZL, CS-905) being antioxidant compound inhibited mesangial cell proliferation induced by highly concentrated insulin (INS) [44]. INS-increased phosphorylation of extracellular signal-regulated kinase (MAPK/ERK) 1/2 was inhibited by 0.1 *μ*M AZL. At the same concentration AZL blocked intracellular ROS production more effectively than 0.1 *μ*M nifedipine. Azelnidipine inhibits insulin-induced mesangial cell proliferation by inhibiting the production of ROS. Thus, AZL is considered to be administered for diabetic nephropathy.

Evaluation of DHP antihypertensive drug** nifedipine** effects on* Saccharomyces cerevisiae* was recently performed [45]. Surprisingly,** nifedipine** exercised a toxic effect on* Saccharomyces cerevisiae* shown through measuring the following parameters: the cell proliferation, respiratory activity, and the level of some OS biomarkers (CAT, MDA).

Effects of some structurally different 1,4-DHP Ca antagonists (four commercial 4-nitrophenyl 1,4-DHP derivatives:** nimodipine**,** nicardipine**,** nifedipine**, and** niludipine**, two originated from LIOS 1,4-DHPs,** cerebrocrast** and** etaftoron**, as well as* two metabolites* of** cerebrocrast**) were studied on rat spleen lymphocyte activation and proliferation in vitro following stimulation with the mitogens: concanavalin A and recombinant interleukin-2 (IL-2), as well as insulin and insulin antibodies [46]. Ca^2+^ antagonists in a concentration range of 10 *μ*M and higher are known to suppress Ca transport into the lymphocyte cytosol, changing a normal response of lymphocytes to mitogens and antigens and so inhibiting their proliferation, as well as IL-2-induced cell proliferation, and their receptor expression on the surface of lymphocytes without cell cytotoxicity. Contrary results with DHP compounds were found at lower concentrations. Authors concluded that in low concentrations (0.1 *μ*M to 1 nM) the tested 1,4-DHP Ca antagonists, especially** cerebrocrast**, stimulated the process of rat spleen lymphocyte proliferation and DNA synthesis [46].

## 5. Conclusions

Some of the studied DHP compounds showed remarkable proliferation regulation properties alone or in combination with anticancer drug doxorubicin (DOXO). They were found to show different concentration-dependence and selectivity for the tested four cell lines (two normal (HMEC, L929) and two malignant (HOS, HeLa)) treated. Nonlinear dose-activity dependence and even bifunctional effects depending on substance concentration were observed for some DHPs, while most of them suppressed the cell growth if used at high concentration.

Compound** IB-32**, also** AP-12** and** IOS-10003**, has shown promising dual activity, proliferation inhibition on cancer cell line and proliferation stimulating effect on normal cell line. Mentioned compounds comprise long alkyl (or aralkyl) chains. Therefore, further search of the dual acting compounds seems to be perspective.

There was no obvious relationship of antiradical activity of the tested DHPs and their influence on cell proliferation observed. Finally, it can be concluded that well-known antioxidant DHP** diludine** was the least effective DHP used, although it is well known for its numerous beneficial effects, so we assume that some of the other DHPs tested might allow development of novel biomedical remedies.

## Figures and Tables

**Table 1:** Studied 1,4-dihydropyridine derivatives, their chemical structures, molecular weight (*M*_*w*_) values, LD_50_ values (on NIH 3T3, normal mice embryonal fibroblast cells), and antiradical activity (ARA) determined by DPPH assay. The untreated level of the DPPH radical is designated as 100%. Data are presented as mean ± SD.

Number	Compound	Chemical structures	*M*_*w*_	LD_50_ mg/kg	ARA ± SD %
Group I					

(1)	**Diludine**	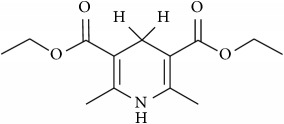	253.30	>2000 (>7.9 mmol/kg) (32,000 mg/kg, mice, ip)	40.5 ± 3.0

Group II					

(2)	**AP-12**	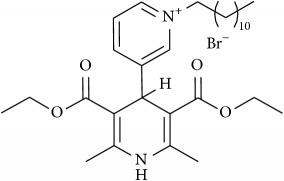	579.61	692	0
(3)	**IB-32**	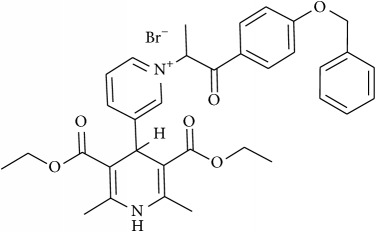	649.57	520	0
(4)	**A2-15 **	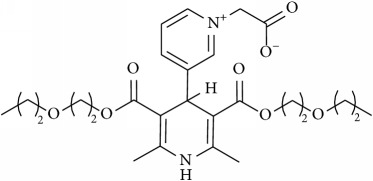	505.56	>2000 (>3.9 mmol/kg)	0

Group III					

(5)	**V-1-32**	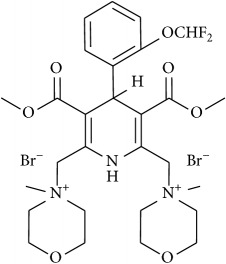	727.43	1706	95.1 ± 0.2
(6)	**IOS-10003**	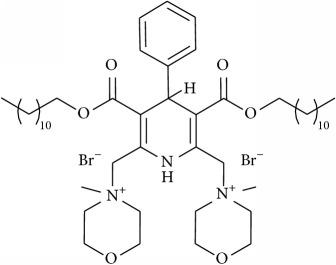	970.01	NA	95.1 ± 0.2
(7)	**IOS-10004**	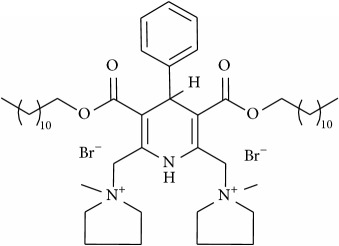	938.01	NA	54.0 ± 0.3
(8)	**V-1-41**	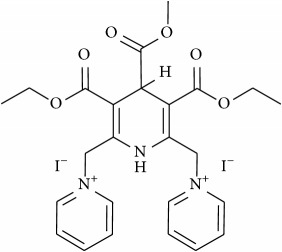	721.32	>2000	70.7 ± 0.9
(9)	**K-2-11**	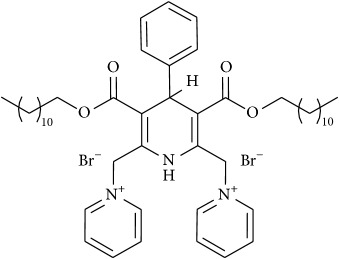	925.91	1482	39.5 ± 0.3
(10)	**D-3-59-1**	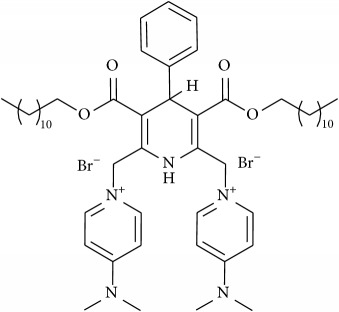	1012.05	1706	27.5 ± 0.2
(11)	**K2-71**	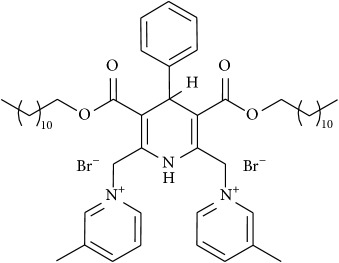	953.98	463 ± 19 (0.9 ± 0.09 mmol/kg)	39.0 ± 2.7
(12)	**Z41-74** [14]	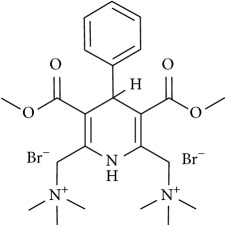	577.35	2425	16.1 ± 0.7

NA: not applicable (the data are absent).

## Abbreviations

DHP(s): 1,4-Dihydropyridine(s)
OS: Oxidative stress
AO: Antioxidant
AOA: Antioxidant activity
ROS: Reactive oxygen species
ARA: Antiradical activity
DOXO: Doxorubicin
pDNA: Plasmid DNA
SIRT1: Sirtuin 1
L929: Murine skin fibroblasts
HMEC: Human mammary epithelial cells
HOS: Human osteosarcoma cells
HeLa: Human epitheloid cervix carcinoma cells
FCS: Fetal calf serum
DMEM: Dulbecco's modified Eagle's medium.
